# Comparison of cytotoxic effects of calcium silicate-based materials on human pulp fibroblasts Mehmet

**DOI:** 10.15171/joddd.2019.037

**Published:** 2019

**Authors:** Mehmet Adıgüzel, Fuat Ahmetoğlu, Ayçe Ünverdi Eldeniz, Mehmet Gökhan Tekin, Bülent Göğebakan

**Affiliations:** ^1^Department of Endodontics, Faculty of Dentistry, Mustafa Kemal University, Hatay, Turkey; ^2^Private Practice, Malatya, Turkey; ^3^Department of Endodontics, Faculty of Dentistry, Selcuk University, Konya, Turkey; ^4^Private Practice, Gaziantep, Turkey; ^5^Department of Biology and Genetics, Mustafa Kemal University, Hatay, Turkey

**Keywords:** Biodentine, cytotoxicity, pulp capping materials, Theracal LC

## Abstract

***Background.*** This study aimed to compare the in vitro cytotoxicity of Theracal LC, BiodentineTM, iRoot BP Plus, and MTA Angelus on human pulp fibroblasts (HPF).

***Methods.*** Fifteen discs from each calcium silicate-based material were prepared in sterile Teflon molds. After setting, the fabricated discs were eluated with a culture medium for 24 h. HPF cells were plated onto 24-well plates at 5×10^3^ cells/well, and the cells were exposed to the material eluates. The cell viability was evaluated with MTT assay at three different times (24, 48, and 72 h). Data were statistically analyzed. The apoptotic/necrotic status of HPF cells exposed to material eluates was determined by flow cytometry.

***Results.*** The differences between the effects of Theracal LC, Biodentine^TM^, MTA Angelus, and iRoot BP Plus on HPF cells were found to be statistically significant (P<0.05). Theracal LC was found to be more cytotoxic considering other vital pulp capping materials at 24- (28.3%), 48- (44.9%), and 72-hour (49.2%) intervals. On the other hand, Biodentine^TM^ showed the least cytotoxic effects (97.1%, 130.0%, and 103.7%, respectively) According to flow cytometry results, Theracal LC material increased apoptosis/necrosis ratios compared to the other materials.

***Conclusion.*** Based on the results of the present study, BiodentineTM, MTA Angelus, and iRoot BP Plus can be classified as biocompatible materials in vital endodontic treatments. However, the Theracal LC materials should be used carefully due to their cytotoxic effects.

## Introduction

The protection of tooth vitality is very important for a favorable dental prognosis. The tooth pulp, which plays a pivotal role in tooth innervation and protection, ensures the production and nutritional content of the dentin.^1^ In addition, the pulp tissue plays a vital role in the production of secondary and reparative dentin.^2^ Although standard root canal treatment is quite successful, it has been reported that the teeth with complex root canal systems and anatomical irregularities have a high failure rate.^3^ Furthermore, when compared to vital teeth, endodontically treated teeth have been suggested to have a greater vulnerability.^4^

Vital pulp therapy includes the placement of biocompatible materials, protection of the health of the exposed pulp using the bioindicator effect, and stimulation of repair via mineralized tissue formation.^5,6^ In this treatment method intended for the protection of pulp health, the applied materials, and the pulp tissue are in contact with each other. Therefore, the degree of toxicity of the material is highly important. These materials, which can be used in pulp capping, perforation repair, and retrograde filling, might adversely affect the vitality of the pulpal and periradicular cells and cause cell death.^7^ Dental materials should help in recovery, stimulate repair for the maintenance of dental function, and be biologically inert.^8^ Therefore, caution should be exercised since the use of materials that are not biocompatible with pulpal and periapical tissue might adversely affect the treatment results.^9,10^

Calcium hydroxide has widely been used in vital pulp therapy. Although this material has useful properties, such as the induction of mineralization, high pH, and low toxicity, some negative properties, such as dissolution over time, mechanical weakness, and the presence of tunnels in the dentin barrier, have also been described.^11^ Mineral trioxide aggregate (MTA), with positive features, such as biocompatibility, and covering and repair capabilities, can be used safely in pulpal and periodontal tissues because of its calcium silicate content.^12^ MTA materials also have some disadvantages; for example, the curing time might take up to four hours. Clinically, its long curing time can lead to many problems, especially in cases where the clinicians must immediately place a restorative agent on the MTA (such as in vital pulp therapy). Overall, the long curing time means more visits for the patient.^13^ Additionally, the inability of the cement to retain its shape under stress, because the yet-to-be-cured material is in direct contact with living tissues, can cause problems with the removal of the cement from the medium.^14^ Therefore, fast-curing calcium silicate-based materials have been suggested for use as pulp capping materials that are in direct contact with the pulp.^15^

Various materials have been produced for use in these treatment methods and marketed. In order to benefit from the favorable properties of the MTA and minimize its disadvantages, some materials, such as Biodentine^TM^ (Septodent, Saint-Maur-Fosses Codex, France), Theracal LC (Bisco Inc., Schaumburg, IL, USA), iRoot BP Plus (Innovative BioCeramix Inc., Vancouver, Canada), Angelus MTA (Angelus, Londrina, PR, Brazil), have been produced. The present study aimed to compare the cytotoxic effects of these materials on HPF. The null hypothesis was that there would be no significant cytotoxic differences between the calcium silicate-based materials.

## Methods

### Cell culture preparation

This study was approved by the Ethics Committee of Mustafa Kemal University. In the present study, HPF cells were obtained from the molar tooth of patients who needed orthodontic treatment. Healthy impacted third molars with dental extraction indication, with no known health problems, were used. Tooth extraction was carried out without damaging the tooth components as far as possible. In order to prevent contamination of experimental materials, the residues, such as gingival tissue attached to the tooth, were removed. Immediately after the extraction, the third molar was cleaned with a piece of gauze soaked with 70% ethanol and then washed with sterile distilled water. The teeth held with maxillary forceps were cut using a sterile fissure bur under water cooling at the cementoenamel junction. Cutting was carried out on the same line, and tooth fragments were placed into the vials containing PBS 1X (Gibco, Carlsbad, US). The samples were transported to the laboratory and placed in Petri dishes in the laminar flow cabinet. All these procedures were performed in 10‒15 minutes. The dental pulp was isolated from the cut tooth fragments by using sterile barbed broach and forceps in the laminar flow cabinet, which is a sterile environment. The cellular separation was completed by digesting the divided pulp tissue with 3 mg/mL of collagenase type I for 60 minutes at 37ºC. The cells were then separated using an insulin syringe and centrifuged for 10 minutes at 1800 rpm. The cell fraction was washed twice with PBS 1X and subjected to centrifugation again for 10 minutes at room temperature at 1800 rpm. The obtained fibroblasts were cultured in DMEM #Hams F12 supplemented with 10% FBS, 2 mL of glutamine, 100 mg/mL of supplemented penicillin G, 100 ug/mL of streptomycin and 1% Fungizone and incubated at 37°C in humidified 95% air and 5% CO_2_ for three weeks. The medium was refreshed every three days until the cells reached 80% confluency.

### Sample preparation

In this study, four calcium silicate-based pulp capping materials, Biodentine^TM^, Theracal LC, iRoot BP Plus, and Angelus MTA, were tested. All the material samples used in the study were prepared in a laminar flow cabinet considering the recommendations of the manufacturers. The surface-to-medium ratio of materials was determined in accordance with ISO 10993-5, and the culture medium was able to completely surround the samples in the release phase. Material discs, measuring 5 mm in diameter and 2 mm in height, were made using cylindrical shaped sterile standard Teflon rings. The initial curing reactions of the samples were completed for cytotoxicity tests.

### MTT assay

Fifteen discs from each group were fabricated for the preparation of material eluates. The prepared test samples were placed in the vials, each containing 15 samples. Cell culture medium was added to the samples in each vial considering surface-to-medium ratio and incubated at 37°C for 24 hours. After the completion of the incubation period, eluates of the materials taken from the vials with a 10-mL syringe and filtered through a sterile filter of 0.22 μm to be used on the cells. HPF cells were plated onto 24-well cell culture dishes, each well with 0.5 mL cell (5000 cells/mL) and incubated at 37°C in the medium with 5% CO_2_ and 95% air for 24 hours. At the end of the incubation period, the cells were exposed to the materials for 24, 48, and 72 hours. The nutrient medium containing only serum was used for the cells in the control group. Eight wells were used for each test material. The viability of the cells exposed to material eluates was evaluated according to succinic dehydrogenase enzyme activity showing the number and activity of cells. After 60 minutes of incubation with 1 mg/mL of MTT, the MTT solution was poured, and 0.5 mL of dimethyl sulfoxide was added to the cells. The change in color was read at 550 nm with a spectrophotometer, which is a colorimetric reader. The assays were repeated at least twice for each test material (n=16).

### Apoptosis Assay

In the present study, a 24-hour exposure period was taken as a basis for apoptosis assay; the apoptotic effects of these materials on the HPF cells were analyzed by comparing with the control group. Sterile material discs were made considering the recommendations of the manufacturer. The prepared test samples were placed into the vials. Cell culture medium was added to the samples in each vial and allowed to stand in the incubator at 37°C for 24 hours. After the completion of the incubation period, the eluates of the materials were taken from the vials with a 10-mL syringe and filtered through a sterile filter of 0.22 μm to be used on cells. HPF cells were placed in wells for the control group and four different test materials. Apoptosis assay was carried out with Annexin V-PE and 7AAD staining in the flow cytometry device by using a fluorescence-activated cell scanner method. Cell apoptosis was performed using the kit containing Annexin V-PE and 7AAD, in line with the recommendations of the manufacturer. The cells were stained simultaneously with Annexin V-PE and 7AAD to distinguish necrotic cells from apoptotic cells. The samples were analyzed using the Cell Quest program.

### Statistical analysis

Statistical analyses were performed using SPSS 18.0 (SPSS Inc., Chicago, IL). The normal distribution of the variables was tested using the Shapiro-Wilk test. One-way ANOVA, post hoc Tukey tests, and unpaired t-test were used to detect any statistical differences between the groups. Each test group was compared with the appropriate control group, and P<0.05 was considered significant.

## Results

**Figure 1:**
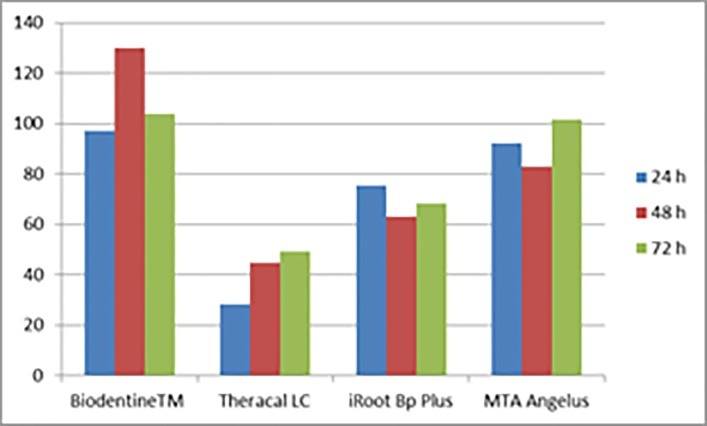
Please insert a caption for the Figure.

Table 1 presents the results of the MTT assay of the calcium silicate-based materials. At 24-, 48-, and 72-hour intervals, the highest cell viability values were observed in Biodentine^TM^ with 97.1%, 130.0%, and 103.7%, respectively, and the lowest viability values were detected in the Theracal LC with 28.3%, 44.9%, and 49.2%, respectively. Theracal LC showed a significantly higher cytotoxic effect compared to the other materials. No significant difference was detected between Biodentine^TM^ and MTA Angelus in terms of cell viability at 24- and 48-hour intervals. These two groups showed significantly higher cell viability than the iRoot BP Plus and Theracal LC materials in the three evaluation periods (Figure 1).

**Table 1:** The cell viability rates of groups

**Time**	**Biodentine** ^TM^	**Theracal LC**	**iRoot BP Plus**	**MTA Angelus**
	**Mean ± SD**	**Mean ± SD**	**Mean ± SD**	**Mean ± SD**
**24 hour**	97.1±3.2	28.3±5.3	75.5±4.9	92.4±20.8
**48 hour**	130.0±7.6	44.9±6.1	63.1±6.4	82.8±2.0
**72 hour**	103.7±2.7	49.2±11.0	68.3±12.9	101.5±35.3

**Figure 2:**
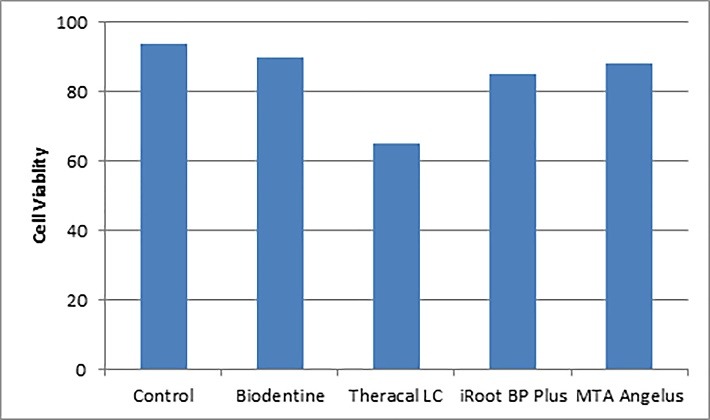
Please insert a caption for the Figure.

The apoptotic effects of calcium silicate-based materials on cell viability are reported in Table 2. The highest viability values were observed in Biodentine^TM^ with 90.48%, and the lowest was detected in the Theracal LC with 69.25%. The Theracal LC showed significantly higher cytotoxic effects compared to the other groups. No significant differences were detected between Biodentine^TM^, iRoot BP Plus, and MTA Angelus in terms of cell viability (Figure 2).

**Table 2:** The apoptotic effect of calcium silicate-based materials on cell viability

	**Control**	**Biodentine** ^TM^	**iRoot BP Plus**	**Theracal LC**	**MTA Angelus**
**Living**	94.45±0.4	90.48±0.4	83.71±1.2	69.25±1.9	85.57±1.4
**Early apoptotic**	0.83±.0.7	2.11±0.5	3.79±0.2	8.70±0.1	3.54±0.3
**Late apoptotic**	4.38±0.4	6.62±0.0.5	6.72±0.4	15.48±1.4	6.76±0.6
**Necrotic**	0.34±0.02	0.79±0.0.1	5.78±0.7	6.57±0.8	4.13±0.6

## Discussion

Biocompatibility is one of the most favorable properties of the materials used in vital endodontic treatments. The biocompatibility of the material is of great importance because of the prolonged contact of the material used in the pulp capping procedure with the surrounding vital pulp tissue.^16^ Dental pulp viability forms the basis of dental function, and biocompatible materials play a pivotal role in repairing the dental pulp exposed after injury, ensuring the regeneration of injured or damaged teeth.

The present study compared the cytotoxic effects of calcium silicate-based materials. The results confirmed that the cell viability values of Theracal LC at both assays were inferior to those of the other materials. Thus, the null hypothesis that there would be no difference in the cytotoxic effect of the tested materials was rejected. A significant reduction was observed in the number of viable cells in the culture medium containing the Theracal LC samples. This is likely due to the unreacted compounds released from the Theracal LC material or the resin content structure. However, in a study conducted by Camilleri et al^17^ on the release of calcium hydroxide from pulp capping materials, it was reported that there was a directly proportional relationship between the release of calcium hydroxide and pulp tissue regeneration. Depending on the completion of hydration in Theracal LC, it was reported that sufficient calcium hydroxide was not produced in the material; therefore, a low rate of release of calcium hydroxide occurred in the material. Based on the results of the present study, we believe that the cytotoxic effects in the Theracal LC material are due to the low total percentage of Portland cement in its content, and the inadequate wetting of the calcium silicate powder.

Zakerzadeh et al^18^ evaluated the cytotoxic effects of Theracal LC, Biodentine^TM^, and ProRoot MTA on HPF cells, reporting that all the materials yielded similar results in terms of cell viability. This result is not consistent with the results of the present study. The cell viability depends significantly on the concentration of the eluate.^16^ Different concentrations might have affected the results of studies. Poggio et al^19^ investigated the cytotoxic effects of different capping materials (Dycal, Calcicur, Calcimol LC, Theracal LC, MTA Angelus, and Biodentine^TM^) on MDPC-23 cells and reported that Biodentine^TM^ showed higher cell viability than the control group at all the time intervals. Collado-González et al^20^ compared the cytotoxicity of several pulpotomy materials on stem cells and reported that Biodentine exhibited better cytocompatibility than MTA Angelus and Theracal LC. The results of these studies are consistent with those of the present study. The results of the present study showed that Biodentine^TM^ had higher cell viability than the control group at all the evaluation intervals. We believe that the increase in cell viability with this material is due to the composition of the Biodentine^TM^, with no impure elements in its structure, as stated by the manufacturer.

Shi et al^21^ compared the cytotoxic effects of the iRoot BP Plus and MTA materials on human gingival fibroblasts in an in vitro medium, and their cell viabilities were evaluated. The cell viabilities of different concentrations of iRoot BP Plus and MTA were found to be between 77.3% and 113.8%, and no cytotoxic effects were observed in either material. In the present study, iRoot BP Plus showed lower cell viability than the Biodentine^TM^ and MTA Angelus materials, and higher cell viability than Theracal LC in the cell viability evaluation at all the time intervals. The differences in similar studies from the literature are believed to be due to the different cell types used.

According to the flow cytometry results of the present study, while the Theracal LC showed higher cytotoxic effects than the other materials, low cytotoxic effects and high cell viability were observed in the Biodentine^TM^, iRoot BP Plus, and MTA Angelus. The iRoot BP Plus, which showed mild cytotoxic effects in the MTT method, exhibited non-cytotoxic effects in the flow cytometry method, in which the apoptotic effects were examined. Additionally, the cell viability values that we found using flow cytometry in the iRoot BP Plus group were observed to be higher than those in the MTT results. According to this data, we can claim that the apoptosis results and MTT results are inconsistent. Since flow cytometry method assesses cell death pattern by determining membrane permeability and MTT provides information about cell viability by measuring mitochondrial metabolic activity, the results of the apoptosis and MTT did not show similarity,^16^ which might explain the differences in the present study.

## Conclusion

Based on the results of the present study, Biodentine^TM^, MTA Angelus, and iRoot BP Plus can be classified as biocompatible materials in vital endodontic treatments. However, the Theracal LC materials should be used carefully due to their cytotoxic effects.

## Competing Interests

The authors deny any conflict of interests with regard to the authorship and/or publication of this article.

## Authors’Contributions

MA, FA, and AÜE were responsible for the concept. MA, MGT, and BG analyzed data. MA and BG carried out the formal analysis. MA, FA, AÜE, and BG were responsible for investigation. MA, MGT, and BG designed the methodology. MA, FA, and AÜF were responsible for the supervision. MA, FA, and AÜE prepared the original draft. MA, FA, and AÜE reviewed and edited the final draft.

## Acknowledgements

None.

## Ethics Approval

This study was approved by the Ethics Committee of Mustafa Kemal University.
